# Sequence analysis and structure prediction of type II *Pseudomonas *sp. USM 4–55 PHA synthase and an insight into its catalytic mechanism

**DOI:** 10.1186/1472-6807-6-23

**Published:** 2006-11-01

**Authors:** Habibah A Wahab, Nurul Bahiyah Ahmad Khairudin, Mohd Razip Samian, Nazalan Najimudin

**Affiliations:** 1 Laboratory of Biocrystallography and Structural Bioinformatics, Universiti Sains Malaysia, Penang, Malaysia; 2 School of Pharmaceutical Sciences. Universiti Sains Malaysia, Penang, Malaysia; 3 School of Biological Sciences, Universiti Sains Malaysia, Penang, Malaysia

## Abstract

**Background:**

Polyhydroxyalkanoates (PHA), are biodegradable polyesters derived from many microorganisms such as the pseudomonads. These polyesters are in great demand especially in the packaging industries, the medical line as well as the paint industries. The enzyme responsible in catalyzing the formation of PHA is PHA synthase. Due to the limited structural information, its functional properties including catalysis are lacking. Therefore, this study seeks to investigate the structural properties as well as its catalytic mechanism by predicting the three-dimensional (3D) model of the Type II *Pseudomonas *sp. USM 4–55 PHA synthase 1 (PhaC1_P.sp USM 4–55_).

**Results:**

Sequence analysis demonstrated that PhaC1_P.sp USM 4–55 _lacked similarity with all known structures in databases. PSI-BLAST and HMM Superfamily analyses demonstrated that this enzyme belongs to the alpha/beta hydrolase fold family. Threading approach revealed that the most suitable template to use was the human gastric lipase (PDB ID: 1HLG). The superimposition of the predicted PhaC1_P.sp USM 4–55 _model with 1HLG covering 86.2% of the backbone atoms showed an RMSD of 1.15 Å. The catalytic residues comprising of Cys296, Asp451 and His479 were found to be conserved and located adjacent to each other. In addition to this, an extension to the catalytic mechanism was also proposed whereby two tetrahedral intermediates were believed to form during the PHA biosynthesis. These transition state intermediates were further postulated to be stabilized by the formation of oxyanion holes. Based on the sequence analysis and the deduced model, Ser297 was postulated to contribute to the formation of the oxyanion hole.

**Conclusion:**

The 3D model of the core region of PhaC1_P.sp USM 4–55 _from residue 267 to residue 484 was developed using computational techniques and the locations of the catalytic residues were identified. Results from this study for the first time highlighted Ser297 potentially playing an important role in the enzyme's catalytic mechanism.

## Background

Polyhydroxyalkanoic acids (PHA) represent a complex class of biodegradable and naturally occurring biopolyesters that consist of hydroxyalkanoic acid monomers. They are produced by a wide range of bacteria as energy storage compounds especially during limited nutritional supplies and in the presence of excess carbon source. PHA synthase is the key enzyme that plays the central catalytic role in PHA production. It uses coenzymeA (CoA) thioesters of hydroxyalkanoic acids (HAs) as the main substrates and catalyzes the polymerization of HAs to yield PHA with the concomitant release of CoA [1,2]. Numerous studies have been carried out on these enzymes and they are well characterized at the molecular level [3-5]. They can be distinguished into four types based on the subunit composition and substrate specificities [6,7].

To date, there is no experimentally determined structural information regarding PHA synthase. However, several studies [8,9] demonstrated that this enzyme possesses the α/β-hydrolase fold domain. The predicted three-dimensional (3D) model of Type III PHA synthase was reported using lipase as the template [9]. For Type I and II PHA synthase enzymes, threading models had also been developed [8,10]. Type I *Ralstonia eutropha *PHA synthase (PhaC_Re_) and Type II *Pseudomonas aeruginosa *PHA synthase (PhaC_Pa_) were modeled employing the structure of lipase from *Burkholderia glumae *and mouse epoxide hydrolase as the templates, respectively. A lipase-box like pentapeptide motif was observed in the models and the catalytic triad was found to be located adjacent to each other [9,10]. Further to the catalytic triad identification, another residue His453 has been identified and thought to be important in the catalytic mechanism of the enzyme in the recent type II PhaC_Pa _model. This was confirmed by their mutagenesis studies where they found that His453 could functionally replace one of the catalytic triad's residue (His480) [8]. Thus is it clear that with each model developed, new information was discovered regarding the structure and function of PHA synthase.

Therefore, we are motivated to perform a thorough sequence analysis of this enzyme and to predict the 3D structure of Type II PHA synthase. For this purpose, we chose PhaC1_P.sp USM 4–55 _as a model enzyme as this enzyme was first isolated by our group, with the ultimate aim to discover new insights on its structure and function. This is especially important in order to understand the catalytic behavior of this enzyme. Surprisingly, in our investigation of the 3D structure of PhaC1_P.sp USM 4–55_, an interesting feature was discovered which has never been highlighted or proposed before. We proposed an extension to the existing catalytic mechanism and that Ser297 might also be important in the formation of an oxyanion hole which might be occurring in the catalytic mechanism.

## Methods

### Data mining and Sequence Analysis

The linear chain of PhaC1_P.sp USM 4–55 _protein containing 559 residues [11] was subjected to various sequence analysis on SWISS-PROT [12], PDB [13] and PIR [14] using BLAST [15] and PSI-BLAST[16]. Pair-wise and multiple sequence alignment between PHA synthase Type I, II and III were carried out using LALIGN [17] and CLUSTALW [18] respectively. Superfamily HMM [19] and PSI-BLAST were used to identify any conserved domains or families found in the protein.

### Model Development and Evaluation

Five secondary structure prediction methods were used in this work to obtain the information on the secondary structure: PSIPRED [20], PHD [21], Prof [22] Sspro [23] and Jnet [24]. The amino acid sequence was then threaded to the library of known folds using mGenThreader [25], 3DPSSM [26] and FUGUE [27]. The sequence-structure alignment calculated using the threading methods was then used as the input for the development of the 3D models employing MODELLER6v2 [28]. Optimization was carried out using DISCOVER module and MODELLER6v2. The resulted models were evaluated using PROCHECK [29], Verify 3D [30], PROVE [31], WHAT_CHECK [32] and ERRAT [33]. The secondary structural assignment was carried out using DSSP [34]. All the graphic presentations of the 3D model were prepared using InsightII and DSViewerPro [35].

## Results And Discussion

### Data mining and Sequence Analysis

**Figure 1:**
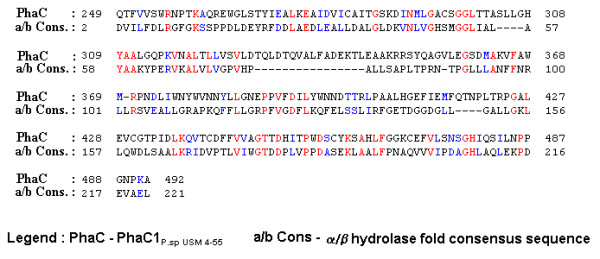
Pairwise sequence alignment between PhaC1_P.sp USM 4–55 _(Gln249 – Ala492) and the consensus sequence of the α/β hydrolase fold family obtained from conserved domain search using BLAST. The alignment showed 32% similarity and 18% sequence identity.

Pair-wise sequence alignment showed that PhaC1_P.sp USM 4–55 _shared 35.1% and 18.9% of sequence identity to both Type I (PhaC_Re_, EMBL: AAZ60717.1) and Type III (PhaC_Cv_, PIR: S29274) enzymes, respectively. Results from BLAST revealed that PhaC1_P.sp USM 4–55 _did not show any significant similarity to any solved 3D structures from PDB (results can be found at [36]). The stretch between residues 249–492 of this enzyme belongs to the α/β hydrolase fold family as demonstrated by the conserved domain search using PSI-BLAST and Superfamily HMM. It was found that 98.2% of these 224 residues of PhaC1_P.sp USM 4–55 _aligned with the α/β hydrolase consensus sequence as shown in Figure 1. The alignment also showed that PhaC1_P.sp USM 4–55 _shared 18% sequence identity and 32% sequence similarity to the α/β hydrolase fold consensus sequence. As the level of identity is very low, the pair-wise alignment described in Figure 1, even if it is suggestive of a potential relationship between the two sequences, may be inaccurate in some regions, thus this has been overcome by performing secondary structure prediction, multiple sequence alignment and threading procedures which will be further discussed below.

**Figure 2:**
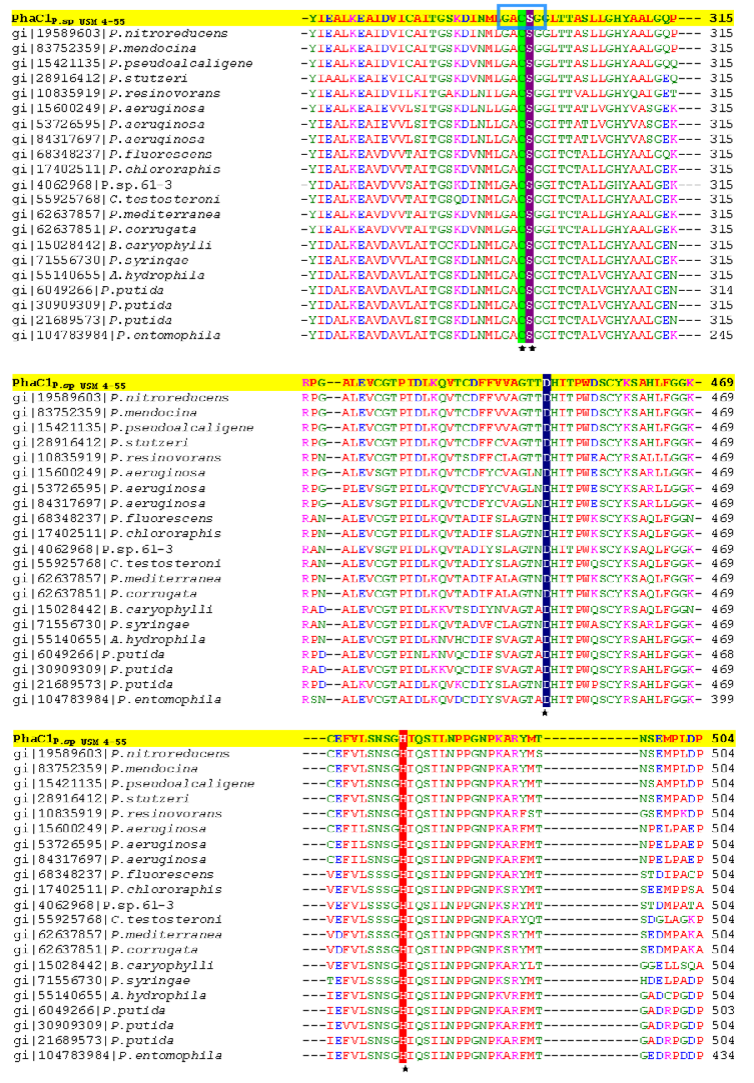
CLUSTALW multiple sequence alignment for Type II PHA synthases with the highly conserved catalytic residues shown in colored blocks; green (Cys296); purple (Ser297); dark blue (Asp451) and red (His479). The consensus pentapeptide motif for PhaC1_P.sp USM 4–55_, which comprises of GACSG is highlighted in the blue box.

A few conserved blocks were identified from the CLUSTALW multiple sequence alignment of various PHA synthases that might play important roles in the enzyme functional properties as shown in Figure 2 (results can be found at [36]). A putative active-site cysteine was identified within a consensus pentapeptide motif made up of "Gly-Xaa-(nucleophile)-Xaa-Gly" frequently found in the active site of the α/β hydrolase fold. This lipase-box like region (GACSG) is located at residue 294 to residue 298 with Cys296 as the nucleophile. This region is one of the conserved region found in all the PHA synthases and the presence of this consensus motif supports the fact that this enzyme conforms to the paradigm of the α/β hydrolase fold. Another residue Ser297, located in the pentapeptide motif next to Cys296, is also highlighted in Figure 2 and is found to be conserved with the Type II PHA synthase family. This indicates that Ser297 might play an important role in the functional activity of the Type II PHA synthase. In this study, it is proposed that Ser297 is vital in the formation of the oxyanion hole which is another feature of the α/β hydrolase fold protein family. The occurrence of this oxyanion hole is discussed further in the next sections.

Diverged from a common ancestor, the α/β hydrolase superfamily [37] of proteins is one of the largest known which includes synthases, esterases, lipases, transferases, thioesterases, haloperoxidases and many more. All of the enzymes of this family share a common fold and despite their differences in catalytic functions, these enzymes harbor a conserved catalytic amino acid sequence of the following format: nucleophile-acid-histidine, in which the nucleophile and the acid varies. The nucleophile can be cysteine, serine or aspartate, and the acid will be either aspartic acid or glutamic acid while histidine is strictly conserved. The multiple sequence alignment also showed that the conserved catalytic aspartic acid and the catalytic histidine are located at residues 451 and 479 respectively. The importance of these residues were suggested by other site-directed mutagenesis studies on the corresponding residues from other types of PHA synthases [10,38-40]. Based on those findings as well as the sequence analysis, we propose that the catalytic residues for PhaC1_P.sp USM 4–55 _comprise Cys296, Asp451 and His479.

### Secondary Structure Prediction

**Figure 3:**
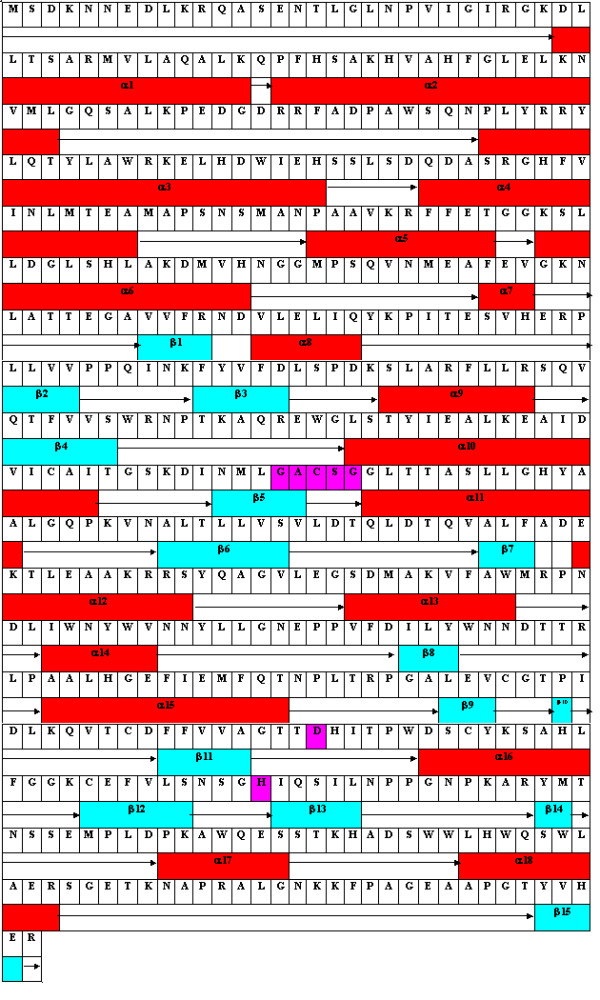
The consensus results of 5 different secondary structure prediction methods for all 559 residues. Red bars represent α-helices, blue bars are β-strands and arrows represent coils. It is predicted that PhaC1_P.sp USM 4–55 _contains 18 α-helices and 15 β-strands. The purple colored boxes are the proposed catalytic machinery for this enzyme.

Results from the secondary structure prediction methods showed that PhaC1_P.sp USM 4–55 _was made up of 18 α-helices and 15 β-strands. These secondary structures were obtained by making a consensus between the results given by the five prediction methods. The consensus structures were shown in Figure 3. The core region which belonged to the α/β hydrolase fold family was observed to contain an alternate pattern of the helices and the strands which complemented with the family fold pattern.

### Template Selection

In the absence of similarity with all solved structures, which could be detected by similarity search programs, threading approach was used to predict the 3D model of PhaC1_P.sp USM 4–55_. In comparative modeling technique, the best template is usually chosen based on the highest percentage of sequence identity between the target and the template proteins. On the contrary, selecting the best template for threading technique is not that straightforward. Sometimes, the proposed templates could turn out to be false positives. The best ranking template does not always thought to be the most suitable template. Due to this drawback as well as the low accuracy of this method, results from the threading programs were carefully examined and compared. The target sequence was scored for its compatibility against the proposed templates as summarized in Table 1. However, only templates with significant high scores were tabulated. All of the templates were found to be in the same family of α/β hydrolase fold.

**Figure 4:**
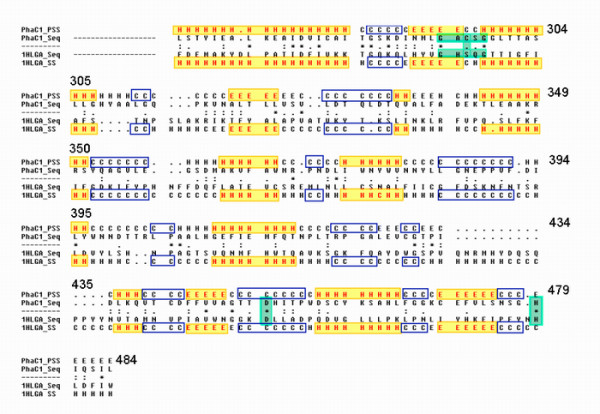
Sequence-structure alignment between PhaC1_P.sp USM 4–55 _(PhaC1_Seq) and 1HLG (1HLGA_seq) generated by 3DPSSM. The filled vertical regions represent the catalytic site for both PhaC1_P.sp USM 4–55 _and 1HLG with their catalytic residues align with each other. The filled boxes represent the aligned secondary structural elements between the two proteins and the blank boxes showed the regions containing loop or random coils also aligned very well between the proteins. (PhaC1_PSS – Consensus secondary structure prediction for PhaC1_P.sp USM 4–55_; 1HLGA_SS – Secondary structure of 1HLG)

Among these templates, only human gastric lipase (1HLG) appeared in all the results given by the three methods. It was either at the top rank or the third best. Having only 11% sequence identity and 42% similarity, 1HLG outperformed the rest of the proposed templates in various ways. Coming from the same family, these two proteins not only shared the same catalytic residues; a nucleophile, an acid and histidine, but it was also found that from the alignment, these residues aligned very well with each other (Figure 4). This was not the case for the other templates in which not all of their catalytic residues aligned with the catalytic residues of the target protein. It was also found that most of the sequence-structure alignments produced between PhaC1_P.sp USM 4–55 _and the other templates contained numerous insertions and deletions that could cause catastrophic problems in the construction of the 3D model. Some templates did show a higher sequence identity compared to 1HLG. For example, 1S80 showed a 14% identity. However, the reliability of the alignment was doubtful due to the reasons described above. Thus, 1HLG was chosen as the modeling template for PhaC1_P.sp USM 4–55_.

For further assessment on the reliability of the structure-sequence alignment between 1HLG and PhaC1_P.sp USM 4–55_, the consensus secondary structure prediction of PhaC1_P.sp USM 4–55 _was used to check the alignment (Figure 4). This was achieved by comparing the aligned secondary structures of 1HLG and the consensus predicted secondary structure of PhaC1_P.sp USM 4–55_. Surprisingly, the structural alignment demonstrated a good alignment of 10 helices, 3 β-strands and 13 random coils altogether. The rest of the regions were shown to have a few structural mismatches and gap-containing segments that most likely will introduce errors in the predicted model.

### 3D Model Building and Evaluation

From the sequence analysis, it was shown that residues which are important in the catalytic activities of the enzyme reside in the α/β hydrolase fold region. Therefore, in order to elucidate the catalytic function of this protein, the 3D structure covering this region was built in this study with the structure sequence alignment between residue 267 to residue 484 of PhaC1_P.sp USM 4–55 _and residue 123 to residue 358 of 1HLG Chain A as shown in Figure 4. The alignment showed an expected result with the conserved catalytic residues of PhaC1_P.sp USM 4–55 _(Cys296, Asp451, His479) aligned with the catalytic triad of 1HLG (Ser153, Asp324, His353). It was also shown that residue Ser297 of PhaC1_P.sp USM 4–55 _aligned with Gln154 of 1HGL which had been agreed to contribute to the oxyanion hole formation for 1HLG [41]. The other regions of the PhaC1_P.sp USM 4–55 _(residue 1 to 266; residue 485 to 559) were not modeled due to unsatisfactory alignments.

**Figure 5:**
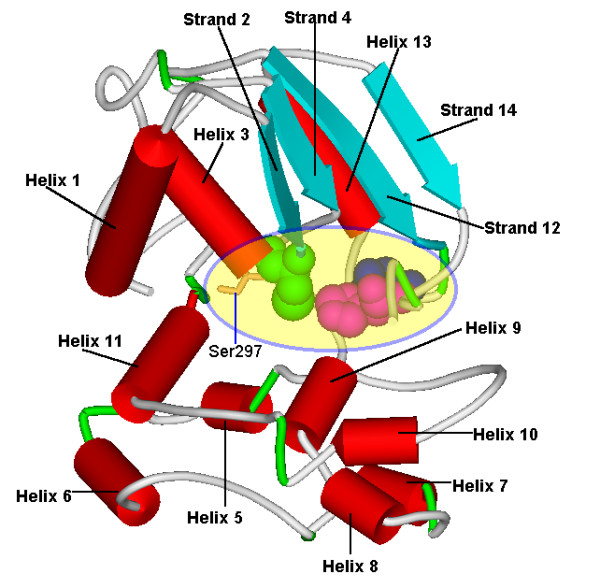
Overall topology of the predicted 3D model of PhaC1_P.sp USM 4–55_. The CPK model represents the catalytic residues, which comprise of Cys296, Asp451and His479 while Ser297 is shown as stick model.

Thirty models comprising all non-hydrogen atoms were generated using MODELLER6v2. The backbone coordinates of the structure 1HLG were assigned to the target sequence according to the structure sequence alignment in Figure 4. Model number 10 was selected as the best model based on the lowest objective function (OF) calculated by the program. MODELLER works based on a satisfaction of spatial restraints. The highlight of the program is to satisfy all the restraints derived from the structure-sequence alignment. Models that produced high violations of the restraints were considered as poor, which in turn lead to higher objective functions, calculated by CHARMM-22 [42] forcefield. The model was then subjected to an optimization scheme to relieve all bad contacts. Eighty models were generated and model number 76 was picked to represent the predicted structure of PhaC1_P.sp USM 4–55 _based on the satisfactory results obtained from evaluation analysis as shown in Figure 5.

**Figure 6:**
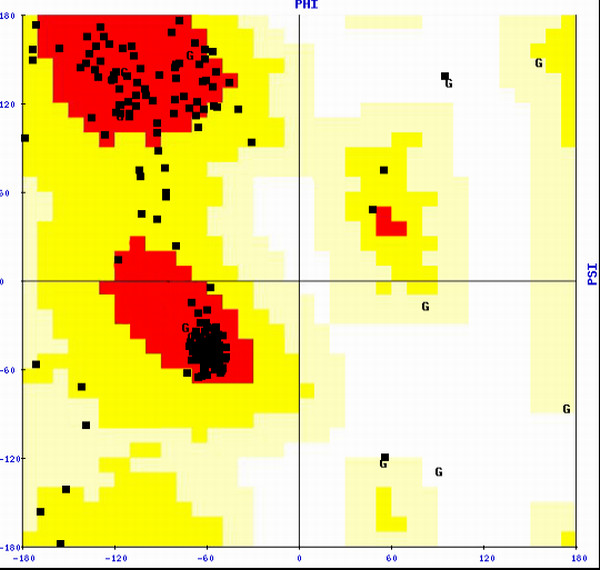
Ramachandran Plot of the 3D model of PhaC1_P.sp USM 4–55_. Red region represents the most favored region, yellow = allowed region, light yellow = generously allowed region, white = disallowed region.

PROCHECK analyses showed that only 2 residues were located in the disallowed region of the Ramachandran Plot and 84.8% of the residues were located in the most favored region (red region in Figure 6). The other residues were found to reside in the additional and generously allowed regions. The two residues that were located in the disallowed region of the Ramachandran plot were Cys296 and Val326. The nucleophile Cys296 was expected to be in the disallowed region due to the sharp and abrupt turn from β-sheet to α-helix which is a conserved and an important feature of the α/β hydrolase fold family. The sharpness of this turn resulted in the phi and psi angles being in an unfavorable region on the Ramachandran plot. As expected, the residues after and before this nucleophile were residues with small side chains (glycine) to ease the packing of the β-strand against the α-helix and to prevent steric conflicts. From the VERIFY_3D analysis, it was found that 82.65% of the residues scored more than 0.2, meaning that 82.65% of the residues complemented with the 1D-3D profile. For the quality of the predicted model to be considered satisfactory, it is expected to have the Verify_3D score of more than 80%. Analysis of the entire structure calculated from PROVE program gave Z-score RMS of 1.746. Z-score above 4.0 and below -4.0 represents the occurrence of many errors in the structure in terms of the packing of the buried atoms. Further analysis was done using the program WHAT_CHECK. It was found that residues Gln330, Asn377, Asn382 and Asn419 have different orientation for their side chains compared to the orientation based on hydrogen bond analysis with solved structures. This might be due to the fact that they could form more energetically favorable hydrogen bonds with their current configuration. Overall, the values obtained from evaluation programs appeared reasonable, taking into account the resolution (3.0Å) of the template and the fact that the structure was obtained by threading with very low sequence identity (11%) and sequence similarity (42%) between the template and the target.

**Figure 7:**
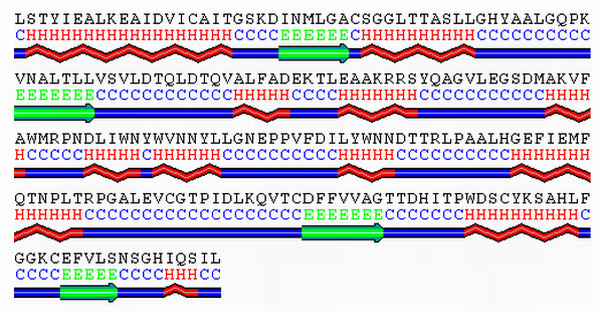
Secondary structure assignment of the predicted three-dimensional model of PhaC1_P.sp USM 4–55 _from residue 267–484 using DSSP. Red ribbon = helices, blue bars = coils and green arrow = strands.

**Figure 8:**
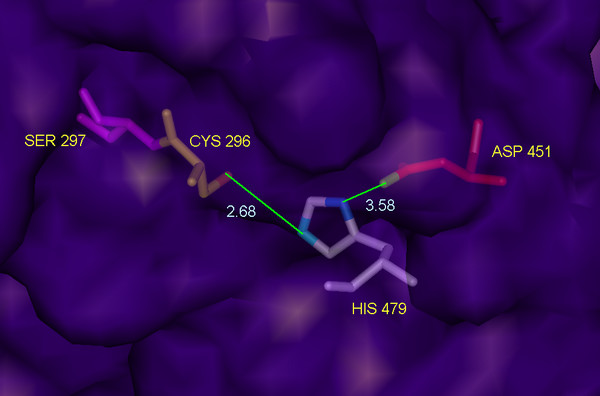
Surface presentation of the active site containing the proposed catalytic residues shown as the stick models (red = Asp451, light purple = His479, orange = Cys296, dark purple = Ser297). These residues are located adjacent to each other and the close proximity between His479 and Cys296 favors the charge relay mechanism.

**Figure 9:**
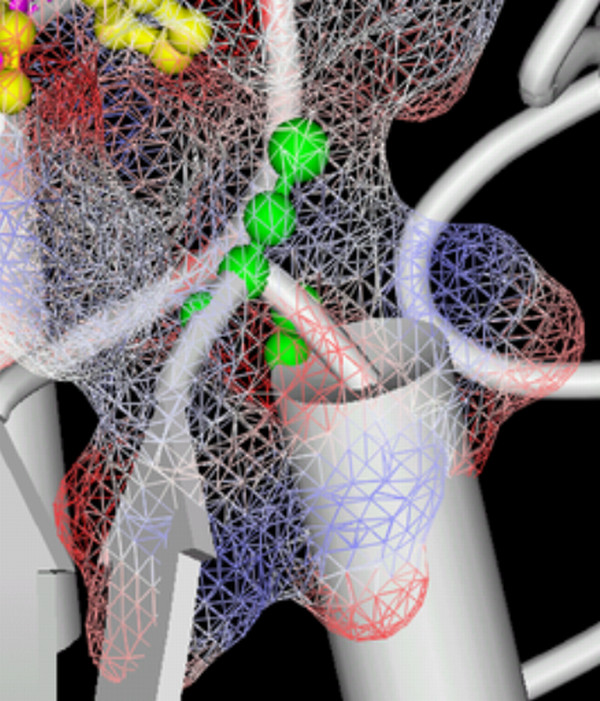
Schematic representation of the 3D model of PhaC1_P.sp USM 4–55 _with the nucleophile Cys296 (green color) located at the sharp turn between β-strand (arrow) and α-helix (cylinder) that results in having unfavorable phi and psi angles. The mesh is colored based on the electrostatic potential around the active site.

The consensus secondary structure prediction methods revealed that regions from residue 267 to residue 484 of PhaC1_P.sp USM 4–55 _consist of 36.2% of α-helices, 16.5% of β-strands and 47.3% of coils. From the developed model, it was found that this region consists of 41.3% of α-helices, 12.4% of β-strands and 46.3% of coils as presented in Figure 7. The two ratios are almost similar demonstrating a good confidence level on the 3D model in terms of the secondary structure formation. As expected, the model showed high similarity to the template structure. The superimposition of the 3D PhaC1_P.sp USM 4–55 _model with the structure of 1HLG yield an RMSD of 1.15 Å covering 86.2% of the backbone atoms. The calculated distance between the Cαs of these residues were such that they had almost the same distance calculated between the Cαs of the catalytic residues of 1HLG as shown in Table 2. Again, this suggested that the threading model was reliable in terms of the accuracy of backbone threading from the template structure. Further inspection of the predicted model revealed that Cys296, His479 and Asp451 were located adjacent to each other at the core structure which was consistent with the work carried out by previous studies [8-10]. Figure 8 shows the specific location for these amino acids. The model also showed that the nucleophile Cys296 was found to reside at the sharp elbow turn from β-strand to α-helix which is a conserved property of the α/β hydrolase fold family [37] as shown in Figure 9.

The resulting model of Type II PhaC1_P.sp USM 4–55 _consists of ten α-helices and four β-sheets which is also similar to the model of Type II PhaC_Pa _developed by Amara and Rehm [8]. Their model showed that the three residues thought to be important in the catalytic activities were identified to be Cys296, Asp452 and His480. This corresponds to Cys296, Asp451 and His279 identified from PhaC1 _P.sp USM 4–55 _3D model. Interestingly, the PhaC1_P.sp USM 4–55 _model revealed another important residue, Ser297, which has not been identified nor discussed before. It is proposed that this residue (highlighted in Figure 5 as well as Figure 8) contribute to the formation of the oxyanion hole and therefore is involved in the catalytic mechanism of PHA synthase. The proposed mechanism will be further discussed in the next section.

### Proposed Catalytic Mechanism

To date, the catalytic mechanism of PHA synthase is still poorly understood. Based on the previous postulated catalytic mechanism initiated by both Steinbüchel and Sinskey Groups [9,10,43,44] and those of Rehm and colleagues [8,10], two subunits of PHA synthase were suggested to form a dimer when active [43]. This dimer will then attach to the surface of the PHA granule for polymerization to take place. It was proposed that during PHA biosynthesis, the thiol group from the first subunit will act as the loading site to load the substrate 3-hydroxyacyl-CoA to the enzyme. The other thiol group from the second subunit will act as the elongation site in which it will be responsible in PHA elongation [44].

From the developed model, the catalytic residues for PhaC1_P.sp USM 4–55 _were believed to consist of Cys296 as the nucleophile, Asp451 as the conserved acid and His479 as the general base catalyst. Thus, we proposed that the catalytic mechanism for PhaC1_P.sp USM 4–55 _specifically and PHA synthase generally, follow the classical catalytic mechanism observed in cysteine and serine proteases [45] since the nature and orientation of the catalytic groups are more or less similar. From Figure 8, the distance between the imidazole group of His479 and the thiol group of Cys296 was calculated to be 3.58 Å, while the distance between the imidazole and the carboxyl group of Asp451 was 2.68 Å. These distances are indeed in the range of distance that are generally found in other α/β hydrolase fold protein such as serine and cysteine proteases as well as lipases that concerns the occurrence of the catalytic triad and the formation of the oxyanion hole [41,46].

**Figure 10:**
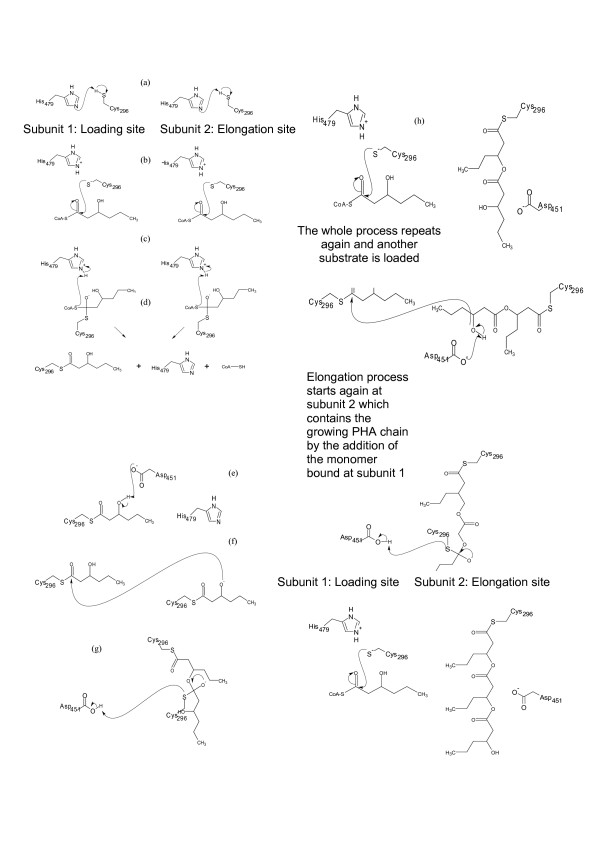
Proposed catalytic mechanism for PhaC1_P.sp USM 4–55 _involving Cys296, Asp451 and His479 with the protein dimer forming the loading and elongation site. In subunit 1 (the loading site), His479 activates Cys296 for nucleophilic attack to the carbonyl carbon of the substrate (3-hydroxyhexanoyl-CoA) yielding a tetrahedral intermediate which then collapses by the release of the CoA group. The elongation process proceeds at the second subunit by the activation of 3-OH group of the bound substrate for nucleophilic attack to the carbonyl carbon of the substrate covalently bound to the Cys296 of the first subunit to form an ester bond. This process repeats by adding more and more monomers to the growing PHA chain.

The complete reaction mechanism for PhaC_P.sp 4–55 _was shown in Figure 10. The close proximity between His479 and Cys296 of PhaC1_P.sp USM 4–55 _as measured from the model enables a proton transfer from the thiol group of the nucleophile Cys296 to His479 (Figure 10(a)). This activates the nucleophilic attack to the carbonyl carbon of the substrate (3-hydroxyhexanoyl-CoA) that will yield a tetrahedral intermediate as shown in Figure 10(b) and 10(c) respectively. This intermediate will then collapse once CoA is released to form CoASH (Figure 10(d)). For the elongation step, which involved the second subunit, the same reaction as above will take place. The next step as shown in Figure 10(e), is the activation of the 3-hydroxyl group of the bound substrate (nucleophile) at the second subunit by Asp451, which acts as the second general base catalyst [43]. This nucleophile will attack the acylated enzyme at the first subunit (Figure 10(f)). Consequently, a second tetrahedral intermediate will be formed which then collapse with the release of Cys296 as the preparation for the next substrate loading (Figure 10(g)). This whole process will repeat as elongation of the PHA chain proceeds (Figure 10(h)).

In contrast to the previous postulated mechanism [8,9,43], the above mechanism accounts for the formation of two tetrahedral intermediates (sp^3 ^hybridization) during the PHA biosynthesis. These transition state intermediates have been widely agreed to be present in all cysteine proteases and lipases [45,47,48] but have not been suggested before in PHA biosynthesis. A vital point to consider here is whether or not this enzyme contains another active site known as the oxyanion hole. This is an important conserved feature of the α/β hydrolase fold protein that is present to stabilize the tetrahedral intermediate state. For the enzyme to work effectively, this unstable transition state needs some form of stabilization. An oxyanion hole is said to occur during the stabilization of the highly negatively charged oxyanion (O^-^) of the tetrahedral intermediate by two hydrogen bonds from the surrounding amide (NH) group such that the NH groups are pointing towards the oxyanion [37,45,47,49]. Several studies have demonstrated that the presence of the oxyanion hole is significant in stabilizing the tetrahedral intermediate in various proteins [46,50,51]. For α/β hydrolase fold proteins, the mandatory feature of the occurrence of the oxyanion hole is that one of the residues that is involved in the hydrogen bonding with the oxyanion is always located next to the nucleophile (Ser297 in PhaC1_P.sp USM 4–55_). From the 3D model, it is very clear that Ser297 could contribute its NH group to stabilize the negatively charge oxyanion formed by the enzyme-substrate complex due to its close proximity to the nucleophile. The orientation of this residue can be observed in Figure 7. Our proposed role of Ser297 in the stabilization of the tetrahedral intermediate could explain why serine is conserved with almost all of the Type II PHA synthase. However, the second NH group that is involved in the stabilization of the tetrahedral intermediate in PhaC1_P.sp USM 4–55 _could not be identified due to the lack of information on the orientation of the enzyme-substrate complex. Perhaps knowledge of how the enzyme interacts with the substrate would probably shed some light into identifying the second residue that contributes to the formation of the oxyanion hole if this feature is indeed present in the PHA synthase catalytic mechanism.

## Conclusion

We have performed sequence analysis and attempted to predict the 3D structure of PhaC1_P.sp USM 4–55 _using the method of fold recognition due to very low similarity to any available experimentally solved 3D protein structures. A series of molecular modeling and computational methods were combined in order to gain insight into the 3D structure of PhaC1_P.sp USM 4–55 _concentrating on the α/β hydrolase fold region. Human gastric lipase was used as the modeling template and from the developed model, it was shown that the catalytic residues were located adjacent to each other. From the sequence analysis and the deduced model, we have also identified Ser297, which we proposed to be involved in the catalytic activity by forming an oxyanion hole. This finding has never been proposed by any other studies before. We also proposed an extension to the catalytic mechanism of this enzyme based on that of serine and cysteine proteases. Two tetrahedral intermediates were postulated to occur during the PHA biosynthesis and we believe that the formation of an oxyanion hole is integral to the catalytic mechanism of this enzyme.

## Figures and Tables

**Table 1:** Proposed template structures obtained from three threading methods along with the calculated scores.

**Method**	**Template**	**Fold**	**% id**	**Score**
**3DPSSM**	Human Gastric Lipase (PDB id: 1HLG)	α/β hydrolase fold	11	E value^a ^= 0.00888
	Human soluble epoxide hydrolase (PDB id: 1S8O)	α/β hydrolase fold	14	E value = 0.327
	Bromoperoxidase (PDB id: 1BRT)	α/β hydrolase fold	11	E value = 0.519
**mGenThreader**	Haloalkane Dehalogenase (PDB id: 1BN6:A)	α/β hydrolase fold	11	E value^b ^= 3e-05
	Mouse Epoxide Hydrolase (PDB id: 1CR6:A)	α/β hydrolase fold	8	E value = 3e-05
	Human Gastric Lipase (PDB id: 1HLG:A)	α/β hydrolase fold	11	E value = 3e-05
**FUGUE**	Human Gastric Lipase (PDB id: 1HLG:A)	α/β hydrolase fold	11	Z-SCORE^c ^= 7.86
	Hydroxynitrile-Lyase (PDB id: 1QJ4)	α/β hydrolase fold	11	Z-SCORE = 6.31
	Proline Iminopeptidase (PDB id: 1AZW)	α/β hydrolase fold	12	Z-SCORE = 5.08

^a ^E-value must be as low as possible

^b ^E-value < 0.001; very significant and certain

^c ^Recommended cutoff : Z-SCORE >= 6.0 (CERTAIN 99% confidence)

**Table 2:** Comparison of the Cα distances for the catalytic residues between the template structure 1HGL and the predicted structure of PhaC1_P.sp USM 4–55_

**Catalytic Residues**	**PhaC1**_**P.sp USM 4–55**_	**1HLG**
**Nucleophile – Histidine**	Cys296 – His479 = 7.95 Å	Ser153 – His353 = 7.41 Å
**Histidine – Acid**	His479 – Asp451 = 4.74 Å	His353 – Asp324 = 4.67 Å
